# Significant gene expression differences in histologically “Normal” liver biopsies: Implications for control tissue

**DOI:** 10.1002/hep.22411

**Published:** 2008-09

**Authors:** Tarik Asselah, Ivan Bièche, Ingrid Laurendeau, Michelle Martinot-Peignoux, Valerie Paradis, Dominique Vidaud, Dominique-Charles Valla, Pierre Bedossa, Patrick Marcellin, Michel Vidaud

**Affiliations:** 1 Service d'Hépatologie & Institut National de la Santé et de la Recherche Médicale (INSERM) U733, CRB3, Université Paris Diderot, Assistance Publique-Hôpitaux de Paris, Hôpital BeaujonClichy, France; 2 INSERMU745, Paris, France; Université René DescartesParis, France; 3 Service de Génétique Moléculaire, Hôpital BeaujonClichy, France; 4 Service d'Anatomie Pathologique, Hôpital BeaujonClichy, France

## Abstract

Gene expression technologies allow the analysis of gene networks whose expression is associated with specific pathological conditions compared with normal tissue. We hypothesized that histologically normal tissue obtained in different ways (percutaneous or surgical liver biopsies), usually used as normal controls in gene expression studies, could have different gene expression patterns. Group A comprised percutaneous liver biopsies in 14 patients with mildly elevated alanine aminotransferase in whom all causes of liver disease had been ruled out. Group B comprised 14 surgical liver biopsies of nontumoral livers. All 28 specimens were histologically normal. Real-time quantitative reverse-transcription polymerase chain reaction were used to compare the messenger RNA expression of 240 selected genes in these two groups. Expression of 26 of the 240 genes was significantly different between groups A and B; 23 genes were up-regulated in group A, while three were down-regulated in group B. The most notable changes occurred in the inflammatory response family genes. Eight genes discriminated perfectly between groups A and B: seven up-regulated genes (*PAI1, THBS1, IL8, PTGS2, CXCR4, JUN*, and *FOS*), and one down-regulated gene (*IHH*). In chronic hepatitis C liver samples, a lower or higher expression of a *IL8* was found depending on whether the controls were obtained percutaneously or surgically. *Conclusion:* Our study demonstrates that histologically normal liver tissue obtained in two different ways (percutaneous or surgical) has different gene expression patterns emphasizing the importance of an adequate selection of histologically normal controls to prevent discordant results in gene expression studies. (Hepatology 2008.)

Gene expression profiling technologies are used to analyze gene networks whose expression is associated with specific pathological conditions compared with normal tissue.^1^ For instance, in 1999, the high expression of a specific group of genes was identified in highly proliferative breast tumor cells that were compared with normal breast tissue samples.^2^

The development of effective tools for large-scale gene expression analysis has already provided new insights into the involvement of gene networks and regulatory pathways in various tumoral processes.^3^ Complementary DNA microarrays can be used to test the expression of thousands of genes at once, while real-time reverse-transcription polymerase chain reaction (RT-PCR) offers more accurate and quantitative information on smaller numbers of selected candidate genes.^4^–^6^

We hypothesized that the histologically normal tissue usually used as normal controls in gene expression studies obtained in two different ways (that is, percutaneous or surgical liver biopsies), might have different gene expression patterns. We suspected that an acute gene response might be observed during surgery because of aggression and stress, despite the absence of any macroscopic injury. To confirm this hypothesis, real-time quantitative RT-PCR was used to quantify the messenger RNA (mRNA) expression of a large number of selected genes in pooled A (histologically normal tissue obtained percutaneously) specimens compared with pooled B (histologically normal tissue obtained surgically) specimens. The expression level of 240 genes known to be involved in various cellular and molecular mechanisms associated with response to stress was examined. We especially focused on the expression of genes related to early stress response, hypoxia, and inflammation.^7^–^12^

Genes of interest were further investigated in 14 individual group A specimens compared with 14 individual group B specimens. We then investigated whether the choice of histologically normal controls could lead to discordance or misinterpretation of specific pathological conditions such as chronic hepatitis C.

## Materials and Methods

We selected liver samples on the basis of a histologically normal pattern: no portal or lobular inflammation and/or necrosis; absence of portal, central, or perisinusoidal fibrosis; and no other significant abnormal features (steatosis <5%, no iron overload, no ballooning or liver cell clarification, no cholestasis or bile duct lesion).

### Group A

**Fig. 1:**
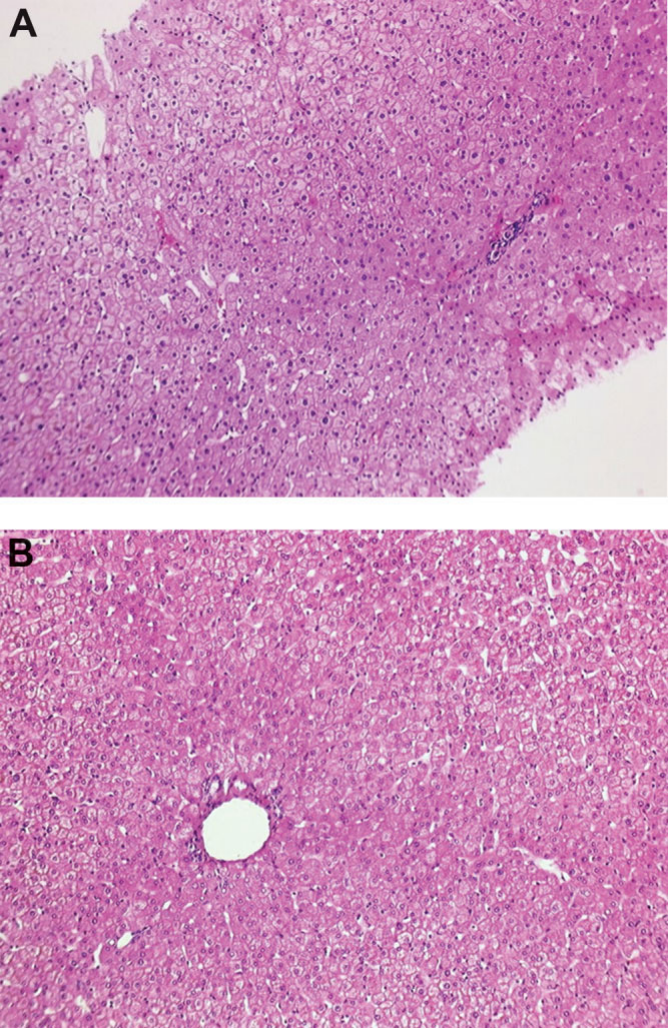
(A) Transparietal biopsy of normal liver. The normal portal tract and central vein are shown. Hepatocytes are arranged in regular trabeculae (hematoxylin-eosin staining; magnification ×25). (B) Surgical sample of normal liver. The lobular architecture is well-preserved; the normal portal tract is present in the center (hematoxylin-eosin staining; magnification ×25).

Group A comprised percutaneous normal liver biopsy specimens, obtained from 14 adults with mildly elevated serum alanine aminotransferase activity addressed to Beaujon Hospital (Clichy, France), in whom all causes of liver disease had been ruled out (medication, alcohol, chronic viral hepatitis, autoimmune processes, and metabolic disease). In these adults, liver biopsies were performed percutaneously under local anesthesia. A transparietal biopsy of a normal liver is illustrated in Fig. 1A.

### Group B

Surgical liver biopsies of nontumoral livers were obtained from 14 adults during operations for liver metastasis of colorectal cancer (n = 7) or benign liver tumors (n = 7) under systemic/general anesthesia. For the purpose of this study, we sampled tissue fragments at least 3 cm from the nearest metastasis. Neither fragment showed portal distorsion or expansion, ductular proliferation, or cholestasis that could suggest a mass effect. A surgical biopsy of a normal liver is illustrated in Figure 1B.

All 28 liver tissue specimens from group A and group B were histologically normal (absence of inflammation, fibrosis, and pathological pattern).

For all cases, one fragment was frozen and used for mRNA extraction and another was formalin-fixed and paraffin-embedded. All these samples were carefully reviewed by two liver pathologists and considered normal.

### Chronic Hepatitis C Patients

Percutaneous liver biopsy specimens obtained from 55 chronic hepatitis C patients, selected from a cohort of untreated patients with chronic hepatitis C followed at Beaujon Hospital (Clichy, France), were graded and staged (Metavir),^13^ and the gene expression was studied (A1F1 [n = 11], A2F1 [n = 9], A1F2 [n = 10], A2F2 [n = 10], A2F3 [n = 15]).

The study was approved by the local ethics committee and conformed to the 1975 Declaration of Helsinki. All patients gave informed consent prior to liver biopsy.

### Large-Scale Real-Time RT-PCR

#### Theoretical Basis

Reactions are characterized by the point during cycling when amplification of the PCR product is first detected, rather than the amount of PCR product accumulated after a fixed number of cycles. The larger the starting quantity of the target molecule, the earlier a significant increase in fluorescence is observed. The parameter Ct (threshold cycle) is defined as the fractional cycle number at which the fluorescence generated by SYBR green dye–amplicon complex formation passes a fixed threshold above baseline. The increase in fluorescent signal associated with exponential growth of PCR products is detected by the laser detector of the ABI-Prism 7900 Sequence Detection System (PerkinElmer Applied Biosystems, Foster City, CA), using PE Biosystems analysis software according to the manufacturer's instructions.

The precise amount of total RNA added to each reaction mix (based on optical density) and its quality (that is, lack of extensive degradation) are both difficult to assess. We therefore also quantified transcripts of two endogenous RNA control genes involved in two cellular metabolic pathways, namely *TBP* (Genbank accession number NM_003194), which encodes the TATA box-binding protein (a component of the DNA-binding protein complex TFIID), and *RPLP0* (also known as 36B4 [Genbank accession number NM_001002]), which encodes human acidic ribosomal phosphoprotein P0. Each sample was normalized on the basis of its *TBP* (or *RPLPO*) content.

Results, expressed as N-fold differences in target gene expression relative to the *TBP* (or *RPLPO*) gene, and termed Ntarget, were determined as Ntarget = 2^ΔCt_sample_^, where the ΔCt value of the sample was determined by subtracting the average Ct value of the target gene from the average Ct value of the *TBP* (or *RPLP0*) gene.

The Ntarget values of the samples were subsequently normalized such that the median value of the percutaneous normal liver specimen Ntarget was 1.

#### Primers and Controls

**Fig. 2:**
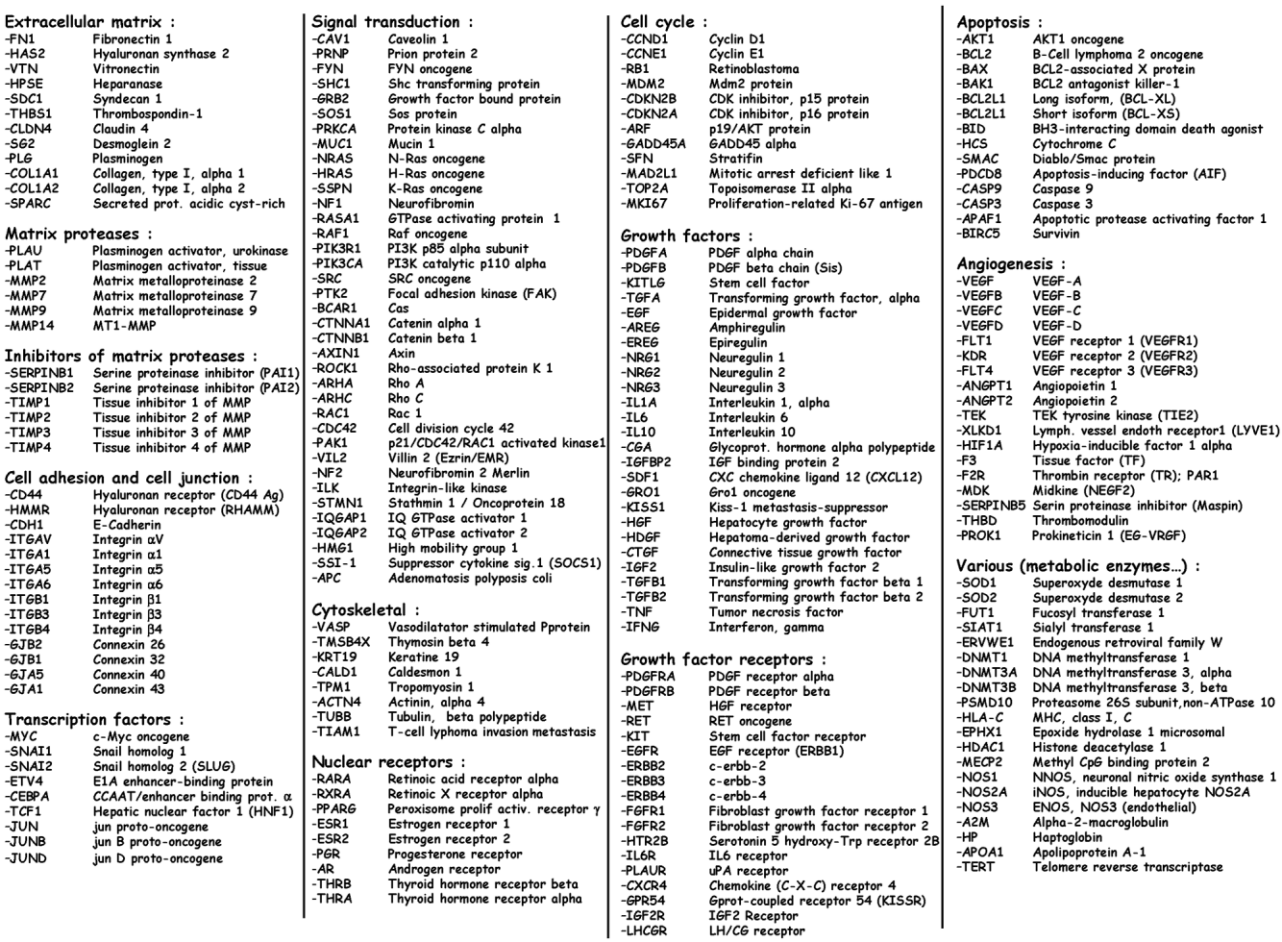
List of the 240 genes studied.

We suspected that, during surgery, as during aggression or stress, an acute gene response would be observed despite the absence of macroscopic injury. Based on a study of the literature describing early gene expression changes during aggression (associated with stress), we selected 240 genes involved in various cellular and molecular mechanisms associated with response to stress and during hepatic stellate cell activation, because these cells participate in the remodeling of injured livers.^7^–^12^ These genes encode proteins involved in the immune response, extracellular remodeling, oxidative stress, signal transduction pathways, cell cycle control, apoptosis, angiogenesis, interferon signaling, and so forth. Approximately 10 to 20 genes were selected per pathway (Fig. 2).

Primers for *TBP*, *RPLP0*, and the 240 target genes were chosen with the assistance of the Oligo 5.0 computer program (National Biosciences, Plymouth, MN).

We conducted searches in the dbEST and nr databases to confirm the total gene specificity of the nucleotide sequences chosen as primers and the absence of single nucleotide polymorphisms. In particular, the primer pairs were selected to be unique relative to the sequences of closely related family member genes or of the corresponding retro-pseudogenes. To avoid amplification of contaminating genomic DNA, one of the two primers was placed at the junction between two exons, if possible. In general, amplicons were between 70 and 120 nucleotides long. Gel electrophoresis was used to verify the specificity of PCR amplicons.

For each primer pair, we performed no-template control and no-RT control (RT-negative) assays, which produced negligible signals (usually >40 in Ct value), suggesting that primer–dimer formation and genomic DNA contamination effects were negligible.

#### RNA Extraction

Total RNA was extracted from frozen liver tissue samples using the acid-phenol guanidinium method. The quality of the RNA samples was determined via electrophoresis through agarose gels and staining with ethidium bromide, the 18S and 28S RNA bands being visualized under ultraviolet light.

#### Complementary DNA Synthesis

Total RNA was reverse-transcribed in a final volume of 20 μL containing 1× RT buffer (500 μM each deoxyribonucleotide triphosphate, 3 mM MgCl_2_, 75 mM KCl, 50 mM Tris-HCl [pH 8.3]), 20 U RNasin ribonuclease inhibitor (Promega, Madison, WI), 10 mM dithiothreitol, 100 U Superscript II ribonuclease H reverse transcriptase (Invitrogen, Cergy Pontoise, France), 3 μM random hexamers (Pharmacia, Uppsala, Sweden), and 100 ng total RNA. The samples were incubated at 20°C for 10 minutes and 42°C for 30 minutes, and reverse-transcription was inactivated by heating at 99°C for 5 minutes and cooling at 5°C for 5 minutes.

#### PCR Amplification

All PCR reactions were performed using an ABI-Prism 7900 Sequence Detection System (PerkinElmer Applied Biosystems) and the SYBR Green PCR Core Reagents kit (PerkinElmer Applied Biosystems). Ten microliters of diluted sample complementary DNA (produced from 2 ng of total RNA) was added to 15 μL of the PCR master mix. The thermal cycling conditions comprised an initial denaturation step at 95°C for 10 minutes, and 50 cycles at 95°C for 15 seconds and 65°C for 1 minute.

### Strategy of Analysis

First, two pools of five liver specimens from each group were respectively constituted by mixing aliquots of equivalent amounts of RNA from each of the liver samples. We then determined the mRNA expression level of the 240 genes in each pool. Genes whose expression differed between pools by at least three-fold in group B versus group A were selected. This robust selection criterion ensures the identification of genes of marked interest.

The expression level of these selected genes was then assessed in each of the 28 individual samples. Comparison of the pool values with the mean individual values showed that RNA pooling was an appropriate initial screening approach, significantly limiting the required number of PCR experiments. Using the same approach, we have previously shown the involvement of several altered molecular pathways in the genesis of hepatitis C virus (HCV) infection,^4^ breast cancer,^14^ and hepatitis C liver fibrosis.^5^

### Statistical Analysis

Relationships between the molecular markers and histological parameters (in both group A and group B and in chronic hepatitis C) were tested using the nonparametric Mann-Whitney *U* test.^15^ Differences between the two populations were judged significant at confidence levels above 95% (*P* < 0.05). To visualize the capacity of a given molecular marker to discriminate between two populations (in the absence of an arbitrary cutoff value), we summarized the data in a receiver operating characteristic (ROC) curve.^16^

The mRNA levels indicated in Tables 1 and 2 (calculated as described in Materials and Methods) show the abundance of the target relative to the endogenous control (*TBP*) to normalize the starting amount and quality of total RNA. Similar results were obtained with a second endogenous control, *RPLP0* (also known as 36B4) (data not shown).

**Table 1:** Significantly Dysregulated Genes in Surgical Nontumoral Liver Patients Relative to Percutaneous Normal Liver Patients

Gene Symbols	Alternate Symbols	Gene Name	Gene Characterization	Percutaneous Normal Liver (n = 14)	Surgical Nontumoral Liver (n = 14)	*P* Value*	ROC- AUC
Significantly up-regulated genes in surgical nontumoral liver patients							
*PAI1*	SERPINE1	Plasminogen activator inhibitor-1	Extracellular matrix	1.0 (0.2-3.6)†	29.7 (9.5-83.9)	0.0000067	1.000
*THBS1*	TPS1	Thrombospondin-1	Extracellular matrix	1.0 (0.3-1.9)	12.4 (5.6-81.2)	0.0000067	1.000
*IL8*		Interleukin-8	Growth factor/cytokine	1.0 (0.6-2.1)	97.9 (3.8-434.7)	0.0000067	1.000
*PTGS2*	COX2	Prostaglandin-endoperoxide synthetase-2	Angiogenesis	1.0 (0.4-1.4)	11.1 (2.5-40.7)	0.0000067	1.000
*CXCR4*		Chemokine (C-X-C motif) receptor-4	Growth factor receptor	1.0 (0.3-1.6)	5.9 (2.1-19.4)	0.0000067	1.000
*JUN*		Jun oncogene	Transcription factor	1.0 (0.2-1.9)	14.0 (3.7-22.5)	0.0000067	1.000
*FOS*		Fos oncogene	Transcription factor	1.0 (0.3-14.8)	57.9 (23.3-220.9)	0.0000067	1.000
*CCL2*	MCP-1	Chemokine (C-C motif) ligand-2	Growth factor/cytokine	1.0 (0.5-2.3)	13.6 (1.2-40.1)	0.000014	0.982
*SOCS3*	SSI-3	Suppressor of cytokine signaling-3 (SSI-3)	Signal transduction	1.0 (0.2-2.4)	28.5 (1.2-71.9)	0.000035	0.959
*CXCL1*	GRO1	Chemokine (C-X-C motif) ligand-1	Growth factor/cytokine	1.0 (0.4-2.2)	9.2 (0,9-56.0)	0.000043	0.954
*HIF1A*		Hypoxia-inducible factor-1, alpha	Angiogenesis	1.0 (0.4-1.5)	2.5 (1.1-6.1)	0.000053	0.949
*MMP9*		Matrix metalloproteinase-9	Extracellular matrix	1.0 (0.3-6.0)	15.0 (0.8-74.2)	0.000094	0.934
*CTGF*		Connective tissue growth factor	Growth factor/cytokine	1.0 (0.1-4.6)	5.4 (0.7-16.8)	0.00020	0.913
*HAS2*		Hyaluronan synthase-2	Extracellular matrix	1.0 (0.5-2.1)	11.0 (0.1-41.8)	0.00024	0.908
*IL6*		Interleukin-6	Growth factor/cytokine	1.0 (0.3-7.9)	58.9 (0.2-338.9)	0.00048	0.888
*EGR1*	KROX-24	Early growth response-1 (KROX-24)	Transcription factor	1.0 (0.2-16.4)	7.1 (2.9-18.9)	0.00048	0.888
*CCL3*	MIP-1A	Chemokine (C-C motif) ligand-3 (MIP-1A)	Growth factor/cytokine	1.0 (0.5-4.0)	6.7 (0.5-21.5)	0.00048	0.888
*CCL4*	MIP-1B	Chemokine (C-C motif) ligand-4 (MIP-1B)	Growth factor/cytokine	1.0 (0.3-2.4)	2.9 (0.3-10.3)	0.0028	0.832
*PAI2*	SERPINB2	Plasminogen activator inhibitor-2	Extracellular matrix	1.0 (0.0-11.4)	26.7 (0.0-165.2)	0.0035	0.824
*CDKN1A*	P21	Cyclin-dependent kinase inhibitor1A (p21 protein)	Cell cycle regulation	1.0 (0.2-3.4)	2.7 (0.5-9.3)	0.0041	0.819
*LIF*		Leukemia inhibitory factor	Growth factor/cytokine	1.0 (0.3-4.2)	5.1 (0.3-17.5)	0.0051	0.811
*CRP*		C-reactive protein	Hepatic secretory protein	1.0 (0.3-14.1)	14.9 (0.4-132.8)	0.0067	0.801
*MMP2*		Matrix metalloproteinase-2	Extracellular matrix	1.0 (0.4-3.0)	1.9 (0.6-24.0)	0.039	0.730
*CXCL5*	ENA78	Chemokine (C-X-C motif) ligand-5	Growth factor/cytokine	1.0 (0.1-18.6)	3.5 (0.3-143,8)	NS	0.702
*COL1A2*		Collagen, type I, alpha-2	Extracellular matrix	1.0 (0.5-2.9)	1.1 (0.1-42.5)	NS	0.594
*IL1A*		Interleukin 1, alpha	Growth factor/cytokine	1.0 (0.0-4.4)	0.9 (0.0-13.0)	NS	0.582
*COL1A1*		Collagen, type I, alpha-1	Extracellular matrix	1.0 (0.0-2.6)	0.8 (0.4-86.4)	NS	0.467
Significantly down-regulated genes in surgical nontumoral liver patients							
*IHH*		Indian hedgehog homolog	Growth factor/cytokine	1.0 (0.28-2.01)	0.06 (0.01-0.20)	0.0000067	0.000
*GPT*		Alanine aminotransferase	Metabolic enzyme	1.0 (0.31-2.78)	0.38 (0.07-1.29)	0.00048	0.112
*IREG1*	SLC11A3, HFE4	Ferroportin-1	Iron metabolism	1.0 (0.55-1.72)	0.54 (0.21-1.66)	0.003	0.171
*CYP2E1*		Cytochrome P450 CYP2E1	Metabolic enzyme	1.0 (0.49-2.47)	0.69 (0.30-1.48)	NS	0.283
*GFAP*		Glial fibrillary acidic protein	Cytoskeletal	1.0 (0.34-8.94)	0.45 (0.22-14.94)	NS	0.298

Abbreviations: AUC, area under the curve analysis; NS, not significant; ROC, receiver operating characteristics.

* Mann-Whitney *U* test.

† Median (range) of gene mRNA levels.

**Table 2:** Genes Perfectly Discriminated Between Percutaneous Normal Liver and Surgical Nontumoral Liver Patients According to Nature of the Adjacent Tumor (Benign Versus Malignant) in the Surgical Nontumoral Group

Gene Symbols	Alternate Symbols	Gene Name	Gene Characterization	Percutaneous Normal Liver (n = 14)	Surgical Nontumoral Liver (n = 14)	Surgical Nontumoral Liver Patients Adjacent to Benign (n = 7)	Surgical Nontumoral Liver Patients Adjacent to Malignant (n = 7)	*P* Value*	ROC- AUC
Genes up-regulated in surgical nontumoral liver patients									
*PAI1*	*SERPINE1*	Plasminogen activator inhibitor-1	Extracellular matrix	1.0 (0.2-3.6)†	29.7 (9.5-83.9)	19.0 (9.5-83.9)	31.1 (9.6-46.5)	NS	0.633
*THBS1*	*TPS1*	Thrombospondin-1	Extracellular matrix	1.0 (0.3-1.9)	12.4 (5.6-81.2)	7.6 (5.6-81.2)	19.9 (9.4-25.7)	NS	0.816
*IL8*		Interleukin-8	Growth factor/cytokine	1.0 (0.6-2.1)	97.9 (3.8-434.7)	80.1 (11.7-434.7)	115.7 (3.8-381.1)	NS	0.388
*PTGS2*	*COX2*	Prostaglandin-endoperoxide synthetase-2	Angiogenesis	1.0 (0.4-1.4)	11.1 (2.5-40.7)	12.9 (5.5-34.4)	9.3 (2.5-40.7)	NS	0.816
*CXCR4*		Chemokine (C-X-C motif) receptor-4	Growth factor receptor	1.0 (0.3-1.6)	5.9 (2.1-19.4)	6.0 (2.1-12.4)	4.2 (2.4-19.4)	NS	0.490
*JUN*		Jun oncogene	Transcription factor	1.0 (0.2-1.9)	14.0 (3.7-22.5)	13.1 (6.2-21.5)	14.9 (3.7-22.5)	NS	0.510
*FOS*		Fos oncogene	Transcription factor	1.0 (0.3-14.8)	57.9 (23.3-220.9)	69.0 (23.3-220.9)	38.6 (29.6-111.3)	NS	0.388
Genes down-regulated in surgical nontumoral liver patients									
*IHH*		Indian Hedgehog homolog	Growth factor/cytokine	1.0 (0.28-2.01)	0.06 (0.01-0.20)	0.07 (0.02-0.20)	0.06 (0.01-0.16)	NS	0.378

Abbreviations: AUC, area under the curve analysis; NS, not significant; ROC, receiver operating characteristics.

* Mann -Whitney *U* test (venign versus malignant).

† Median (range) of gene mRNA levels.

## Results

### mRNA Expression of the 240 Genes in the Group B Pool Sample Relative to the Group A Pool Sample

The mean *TBP* gene Ct (threshold cycle) values for the group A pool and the group B pool were 25.23 ± 0.24 and 25.43 ± 0.23, respectively.

Seven (2.9%) of the 240 genes were detectable but not reliably quantifiable in both the group B and group A pools (Ct > 32). The mRNA expression of 32 (13.7%) of the remaining 233 genes showed at least a three-fold difference between the two pools; 27 (84.4%) genes were up-regulated and 5 (15.6%) were down-regulated in the group B pool sample compared with the goup A pool sample.

### mRNA Expression of the 32 Dysregulated Genes in 14 Group B Samples and 14 Group A Samples

The expression level of the 32 dysregulated genes identified via pooled sample analysis was then determined individually in the 14 group B samples and 14 group A samples.

Twenty-three (85.2%) of the 27 up-regulated genes identified by pooled sample analysis were significantly up-regulated in the 14 group B samples compared with the 14 group A samples (*P* < 0.05; Table 1). Three (60%) of the five down-regulated genes identified via pooled sample analysis were significantly down-regulated in the 14 group B samples compared with the 14 group A samples (*P* < 0.05; Table 1).

The 23 up-regulated genes mainly encoded proteins involved in immune response (interferon pathway, growth factor, growth factor receptor, cytokine: *IL8*, *CXCR4*, *CCL2*, *CXCL1*, *IL6*, *CCL3*, *CCL4*, *LIF*, *CXCL5*, *IL1A*); and matrix remodeling (angiogenesis, extracellular matrix, extracellular matrix protease, inhibitors of matrix protease: *PAI1*, *THBS1*, *PTGS2*, *HIF1A*, *MMP9*, *CTGF*, *HAS2*, *PAI2*, *MMP2*, and *COL1A1*).

The capacity of each of these 26 dysregulated genes (23 up-regulated and 3 down-regulated) to discriminate between group B and group A samples was then tested via ROC curve analysis. The overall diagnostic values of the 26 molecular markers were assessed in terms of their area under the curve (AUC) values (Table 1).

**Fig. 3:**
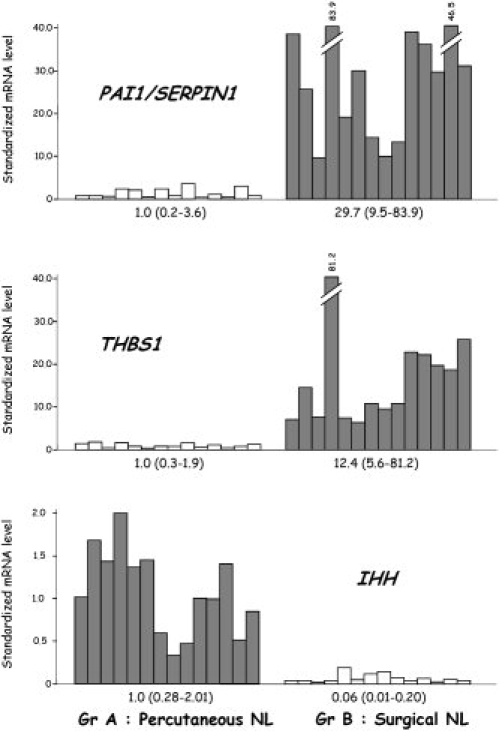
Shown are the mRNA levels of three perfectly discriminatory genes (*PAI1*, *THBS1*, *IHH*) in the 14 percutaneous normal liver samples and the 14 surgical nontumoral liver samples. The median value (range) is indicated for each subgroup. NL, normal liver.

Eight genes perfectly discriminated between groups A and B (AUC-ROC, 1.000): seven up-regulated genes (*PAI1*, *THBS1*, *IL8*, *PTGS2*, *CXCR4*, *JUN*, and *FOS*) and one down-regulated gene (*IHH*). Fig. 3 shows the mRNA levels of three of these genes (*PAI1*, *THBS1*, and *IHH*) in each of the 14 group B samples and the 14 group A samples.

Among the eight genes that discriminated perfectly between the group B and group A samples, there was no significant difference in samples from group B when they were compared for the nature (that is, benign or malignant) of the distant tumor (Table 2).

### mRNA Expression of IL8 in Different Stage of Chronic Hepatitis C in Comparison with Group B Samples and Group A Samples

To determine whether the choice of histologically normal controls could lead to discordance or misinterpretation of specific pathological conditions such as chronic hepatitis C, we measured one (*IL8*) of the eight perfectly discriminating genes in five series of various grades of necroinflammation and stages of liver fibrosis (A1F1, A2F1, A1F2, A2F2, A2F3).

**Fig. 4:**
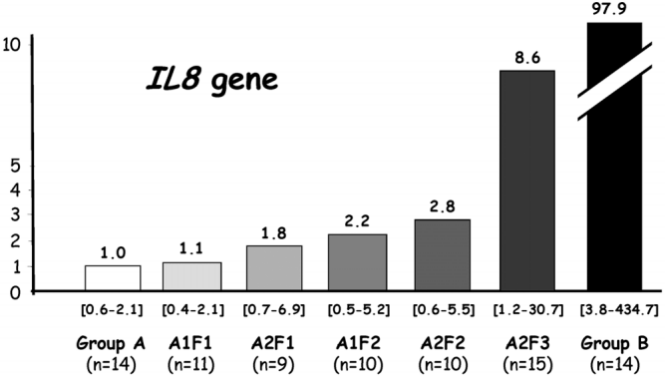
*IL8* expression in different grade of necroinflammation and stage of fibrosis in chronic hepatitis C as compared with group B or group A.

*IL8* was investigated because it has been shown in culture cells that the HCV nonstructural 5A protein induces *IL8*.^17^ *IL8* mRNA expression increases from mild chronic hepatitis C (A1F1) to severe liver lesions (A2F3) (Fig. 4). The results show an underexpression or overexpression of specific genes (such as *IL8*) in HCV infection depending on whether the controls were obtained percutaneously or surgically. It is interesting to note that in this example, group A seems to be the more appropriate control, because an increase in *IL8* mRNA levels from mild (A1F1) to advanced disease (A2F3) is observed, suggesting a model with *IL8* activation during fibrogenesis.

### mRNA Expression of Other Genes Involved in the Hedgehog-Gli Signaling Pathway in Group B and Group A Samples

**Fig. 5:**
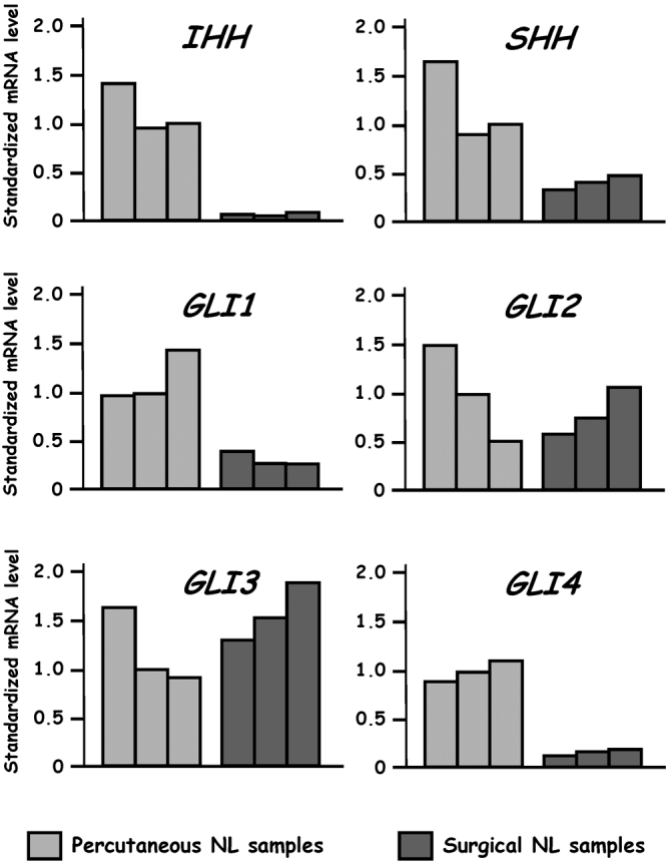
Expression level of Hedgehog/Gli genes in three high *IHH*-expressing percutaneous normal liver samples and three low *IHH*-expressing surgical nontumoral liver samples. NL, normal liver.

The only down-regulated gene that perfectly discriminated between groups A and B (*IHH*) is involved in the Hedgehog-Gli signaling pathway. To further explore the Hedgehog-Gli signaling pathway to discriminate between groups A and B, we tested the expression of six additional genes involved in this pathway (*DHH*, *SHH*, *GLI1*, *GLI2*, *GLI3*, and *GLI4*) in three high *IHH*-expressing percutaneous normal liver samples and three low *IHH*-overexpressing surgical nontumoral liver samples. The results are summarized in Fig. 5. *DHH* transcripts were detectable but not reliably quantifiable in both the group B and group A samples (Ct > 32). Total positive associations (AUC-ROC, 1.000) were found between *IHH* and three of the five expressed genes (*SHH*, *GLI1*, and *GLI4*).

## Discussion

Gene expression profiling technologies are used to analyze gene networks whose expression is associated with specific pathological conditions compared with normal tissue.^1^ Generally, normal tissue for normal controls is obtained in various ways, including percutaneous and surgical biopsy.^1^–^2^,^5^,^18^

This study focused on the gene expression changes observed in the histologically normal liver in relation to the sampling method (percutaneous or surgical liver biopsy). We analyzed the gene transcriptional profiles of percutaneous normal liver specimens, obtained under local anesthesia from 14 adults with mildly elevated serum alanine aminotransferase activity in whom all causes of liver disease had been ruled out (medication, alcohol, chronic viral hepatitis, autoimmune processes, and metabolic disease) compared with nontumoral liver biopsies obtained from 14 adults during surgery for liver metastasis of colorectal cancer or benign liver tumors. All 28 liver tissue specimens (groups A and B) were histologically normal. For our study, we selected liver samples based on a histological normal aspect carefully analyzed by two liver pathologists.

The 26 genes that were significantly dysregulated (23 up-regulated and three down-regulated) in the group B samples mainly encoded proteins involved in immune response (interferon pathway, growth factor, growth factor receptor, cytokine: *IL8*, *CXCR4*, *CCL2*, *CXCL1*, *IL6*, *CCL3*, *CCL4*, *LIF*) and matrix remodeling (angiogenesis, extracellular matrix, extracellular matrix protease, inhibitors of matrix protease: *PAI1*, *THBS1*, *PTGS2*, *HIF1A*, *MMP9*, *CTGF*, *HAS2*, *PAI2*, and *MMP2*). The gene up-regulations in the surgical nontumoral biopsies were not due to tumor cell contamination or stroma cell activation, because similar expression levels were observed in the normal liver samples associated with distant malignant tumors compared with those associated with distant benign tumors.

Most of these genes belong to the acute phase response family and are up-regulated after “stress.”^19^ All living organisms need to sense and respond to conditions that stress their homeostatic mechanisms. The liver plays a central role in the body's response to injury.^20^ Expression of hepatic acute-phase and heat-shock genes probably contributes to restoring homeostasis after surgical procedures. Activation of the acute phase response can be due to different causes, such as hypoxemia, infection, surgery, and anesthesia. The acute phase response gene family includes and/or interacts with numerous family genes (inflammation, cytokines, extracellular matrix, and so forth). Systemic stressors can lead to regeneration.^12^ Hypoxia—a reduction in the normal level of tissue oxygen tension—occurs during acute and chronic vascular diseases, pulmonary disease and cancer.^21^ Another type of hypoxia known as acute or perfusion-limited hypoxia occurs when aberrant blood vessels are shut down, which also causes a reverse in blood flow. Closed vessels can be reopened, leading to reperfusion of hypoxic tissue with oxygenated blood. This leads to an increase in free radical concentrations, tissue damage, and activation of stress-response genes—a process known as reoxygenation injury. It should be noted that dysregulation of *HIF1A*, a gene playing a major role in hypoxia, was observed in this study.

What about surgical liver biopsies under general anesthesia? General anesthetics are known to transiently increase expression of mRNAs of immediate-early genes in the brain.^22^ Furthermore, anesthesia has been shown to mimic ischemic preconditioning,^23^ the process by which brief exposure to ischemia provides robust protection or tolerance against the injurious effects of longer-term ischemia via expression of acute phase response genes.

Among the 26 dysregulated genes identified in this study, eight perfectly discriminated between groups A and B (AUC-ROC, 1.000): seven up-regulated genes (*PAI1*, *THBS1*, *PTGS2*, *CXCR4*, *JUN*, *FOS*, and *IL8*) all involved in the acute phase response, and one down-regulated gene (*IHH*) that codes one of the three mammalian Hedgehog (Hh) proteins playing a major role in vertebrate development and tumorigenesis.

### THBS1, PAI1

*THBS1* and *PAI1* code molecules involved in matrix turnover. Thrombospondins form a family of secreted glycoproteins with pleiotropic functions and widespread expression.^24^–^26^ *THBS1* is involved in the regulation of cellular responses to injury. It has been shown that *THBS1* acts as a strong promoter of transforming growth factor β effects in hepatic stellate cells.^27^ Plasminogen activator inhibitor-1 (PAI-1) is the main physiological inhibitor of both the urokinase-type plasminogen activator and the tissue plasminogen activator and thereby plays an important role in regulation of the fibrinolytic system. PAI-1 has also been reported to act as an acute phase protein,^28^ and plasma PAI-1 levels rise markedly during disease states often associated with an acute phase response, including trauma, surgical procedures, and burn injury. The inflammatory response is a nonspecific reaction of the human body to trauma, injury, or infection, and the liver is a major site for synthesis of inflammatory and procoagulant mediators, including C-reactive protein, fibrinogen, interleukin-6, and PAI-1.^29^

### CXCR4, IL8, and PTGS2

*CXCR4*, *IL8*, and *PTGS2* code molecules involved in angiogenesis and inflammation*.*

Stromal cell–derived factor-1 is a member of the C-X-C motif (CXC) chemokine family that binds to the seven-span transmembrane G-protein–coupled CXCR4 receptor, which has stromal cell–derived factor-1 as its unique ligand.^30^ CXCR4 is expressed by most leukocyte populations, endothelial cells, as well as epithelial and carcinomatous cells. In a recent study, hepatic regeneration was induced by treating rats with 2-acetylaminofluorene and followed by partial hepatectomy.^31^ *CXCR4* mRNA expression, assessed by both quantitative RT-PCR and *in situ* hybridization, was increased during hepatic regeneration.

*PTGS2*, also called *COX-2*, plays an important role in tumor and endothelial cell biology. Increased expression of *PTGS2* occurs in multiple cells within the tumor microenvironment, which can affect angiogenesis. *PTGS2* appears to play a key role in the release and activity of proangiogenic proteins.^32^

Interleukin-8, a cytokine of the CXC chemokine family, plays an important role in tumor progression and metastasis in a variety of human cancers, including lung cancers.^33^ Interleukin-8 biological activity in tumors and the tumor microenvironment may contribute to tumor progression through its potential function in the regulation of angiogenesis, cancer cell growth and survival, tumor cell motion, leukocyte infiltration, and modification of immune responses.

*IL8* mRNA expression increases from mild chronic hepatitis C (A1F1) to severe liver lesions (A2F3). In prior immunohistochemical studies of HCV infection, IL8 protein was shown to be expressed in infiltrating cells in the portal tract and fibrotic septa and within hepatic lobules in patients.^34^ We have previously reported that there was a correlation between intrahepatic mRNA *IL8* expression and hepatic fibrosis in HCV patients.^5^ Moreover, exposure of human umbilical vein endothelial cells to HCV-like particles resulted in increased IL8 production.^35^

### JUN, FOS

The AP-1 transcription factor is mainly composed of Jun, Fos, and/or ATF protein heterodimers. AP-1 mediates gene regulation in response to a plethora of physiological and pathological stimuli, including cytokines, growth factors, stress signals, and bacterial and viral infections, as well as oncogenic stimuli.^36^ Interestingly, a rat model after portal branch ligation produced atrophy of the deprived lobes (70% of the liver parenchyma), whereas the perfused lobes undergo compensatory regeneration; c-fos and c-jun expression were elevated during the first 2 hours in all the compartments.^37^ These findings suggest that the cellular and molecular changes that occur early in a regenerating liver are nonspecific, possibly stress-induced cellular responses. They do not indicate future progression toward atrophy or regeneration.

### IHH and the Mammalian Hedgehog Proteins

Among the 26 dysregulated genes, we identified eight that perfectly discriminated between group A and group B (AUC-ROC, 1.000), one of which is a down-regulated gene (*IHH*) that codes one of the three mammalian Hedgehog (Hh) proteins. Alteration of this unexpected pathway was confirmed via identification of an alteration of additional genes involved in this signaling pathway (one additional ligand [*SHH*] and two transcriptional factors [*GLI*1] and [*GLI*4]). The Hh pathway has been shown to direct the fate of neural and myofibroblastic cells during embryogenesis and during tissue remodeling in adults.^38^–^39^ Recent studies suggest a major role for the Hh pathway in hepatic stellate cell activation and viability^40^ and in the maintenance of hepatic progenitors during fetal development and adulthood.^41^ Fatty liver injury alters Hh activity in liver progenitors, and this might promote epithelial–mesenchymal transitions that result in liver fibrosis.^42^ Hh dysregulation is also observed in human hepatocarcinogenesis.^43^ Our results regarding Hh signaling could suggest qualitative or quantitative variations in hepatic stellate cells and/or hepatic progenitors between percutaneous and surgical normal liver tissues.

This study demonstrates that histologically normal liver tissue obtained in two different ways (percutaneous or surgical liver biopsy) has different gene expression patterns, though all specimens are histologically normal. The most notable changes in gene expression mainly occurred in the inflammatory response gene family. Therefore, this study emphasizes the importance of an adequate selection of histologically normal controls to prevent discordant or false results in gene expression profile analysis.

It is difficult to state which is the best histologically normal control. In any study, the appropriate histological normal control should be obtained in the same technical way as the pathological sample. For instance, in a study of chronic hepatitis C in which liver samples are obtained percutaneously, the histologically normal samples should be obtained through percutaneous liver biopsy.^4^–^6^ In all cases, the controls used should be clearly described. Finally, the careful selection of controls is crucial, since the wrong selection could lead to misinterpretation of results.

## Abbreviations

AUC: area under the curve
Ct: cycle threshold
HCV: hepatitis C virus
Hh: Hedgehog
mRNA: messenger RNA
PAI-1: plasminogen activator inhibitor-1
PCR: polymerase chain reaction
ROC: receiver operating characteristic
RT-PCR: reverse-transcription polymerase chain reaction.
